# Long-acting nitrate use before and after revascularization to evaluate angina in chronic coronary syndrome: a case-crossover study from SCAAR

**DOI:** 10.1016/j.lanepe.2025.101507

**Published:** 2025-10-28

**Authors:** Sacharias von Koch, Tania Sharma, Ramzi Khamis, Tomas Jernberg, Stefan James, Elmir Omerovic, Sammy Zwackman, Johan Sjögren, David Erlinge, Moman A. Mohammad

**Affiliations:** a Department of Cardiology, Clinical Sciences, Lund University, Skåne University Hospital, Lund, Sweden; b National Heart and Lung Institute, Imperial College London, London, United Kingdom; c Department of Clinical Sciences, Danderyd Hospital, Karolinska Institutet, Stockholm, Sweden; d Department of Medical Sciences, Uppsala University, and Uppsala Clinical Research Center, Uppsala, Sweden; e Department of Cardiology Sahlgrenska University Hospital, Gothenburg, Sweden; f Department of Health, Medicine and Caring Sciences and Department of Cardiology, Linköping University, Linköping, Sweden; g Department of Cardiothoracic Surgery, Clinical Sciences, Lund University, Skåne University Hospital, Lund, Sweden

**Keywords:** Chronic coronary syndrome, Angina, Percutaneous coronary intervention, Coronary artery bypass graft surgery, Long-acting nitrates

## Abstract

**Background:**

The ORBITA and ORBITA-2 trials have provided valuable insights into the effects of coronary revascularization in chronic coronary syndrome (CCS). However, uncertainties remain regarding the efficacy of revascularization on symptoms in large real-world populations. To evaluate the efficacy of revascularization, we used dispensed long-acting nitrates as a proxy for the presence of angina.

**Methods:**

The Swedish Coronary Angiography and Angioplasty Registry (SCAAR) was used to identify all patients with CCS and at least one stenosis ≥50% undergoing angiography between the 1st of January 2014 and the 16th of January 2020. Four groups were defined based on treatment strategy: coronary artery bypass graft (CABG) surgery, complete revascularization with percutaneous coronary intervention (PCI), incomplete revascularization with PCI, and no revascularization. As patients in these treatment arms are inherently different, we employed a case-crossover design where each patient served as their own control with data collected during two periods: 1 year before up until angiography and 1–2 years after. This study design inherently controls for time-invariant confounding. The primary outcome was the use of long-acting nitrates defined as a dispensed prescription during the studied periods. Conditional Poisson regression was used to analyse the data.

**Findings:**

For this study, 15,955 patients were eligible. CABG, complete revascularization with PCI, and incomplete revascularization with PCI were associated with a decrease in dispensed prescriptions of long-acting nitrates (from 989/2218 [30.8%] to 156/3207 [4.9%]; risk-ratio (RR): 0.16 [95% confidence interval (CI): 0.13–0.19]), (from 1676/7525 [22.3%] to 966/7525 [12.8%]; RR: 0.58 [95% CI: 0.53–0.62]), and (from 601/2180 [27.6%] to 495/2180 [22.7%]; RR: 0.82 [95% CI: 0.73–0.93]), respectively. No difference was observed for no revascularization (from 864/3043 [28.4%] to 856/3043 [28.1%]; RR: 0.99 [95% CI: 0.90–1.09]).

**Interpretation:**

Revascularization reduces the use of long-acting nitrates in patients with CCS, suggesting angina symptom improvement. CABG appears to provide a more significant effect than PCI, with complete PCI demonstrating greater effectiveness than incomplete revascularization.

**Funding:**

This work was supported by The Swedish Heart and Lung Foundation, ALF, Skane University Hospital funds, the Crafoord Foundation and the 10.13039/501100007308Swedish Medical Association.

**Research in context**

**Evidence before this study**

The symptomatic efficacy of revascularization in chronic coronary syndrome (CCS) has been explored in the ORBITA trials. We searched PubMed from the database inception to November 31, 2024, using the search term (PCI OR “percutaneous coronary intervention” OR CABG OR “coronary artery bypass”) AND angina AND (“stable angina” OR “chronic coronary syndrome”) AND (cohort OR registry OR nationwide OR “population-based”). The symptomatic efficacy of revascularization in chronic coronary syndrome has been explored in the ORBITA trials. Despite the important insights provided by the two ORBITA trials, they had limitations in generalizability leaving questions about the effectiveness of revascularization in larger, unselected, real-world populations.

**Added value of this study**

To address this gap in knowledge, we performed a nationwide case-crossover study leveraging data from the Swedish Coronary Angiography and Angioplasty Registry (SCAAR). Using SCAAR, this analysis included 15,955 patients with CCS. Four groups were defined based on treatment strategy: coronary artery by-pass graft surgery (CABG), complete PCI, incomplete PCI, and no revascularization. We evaluated the use of long-acting nitrates before and after treatment to assess symptomatic benefits. Our findings demonstrate that revascularization, whether by CABG, complete PCI, or incomplete PCI, were associated with a reduction in the use of long-acting nitrates, suggesting improved angina control. In contrast, patients who did not undergo revascularization showed no change in long-acting nitrate use. Importantly, CABG was associated with the largest reduction in dispensed nitrates. This large, real-world study provides new insights into the comparative effectiveness of different revascularization strategies.

**Implications of all the available evidence**

In a large European cohort, revascularization strategies were associated with reduced use of long-acting nitrates. Our findings build on the insights of the ORBITA trials by offering population-level evidence of the symptomatic benefits of revascularization in routine clinical practice. These results have important implications for clinical decision-making by reinforcing the value of revascularization for symptom relief in CCS.

## Introduction

Trials assessing the role of revascularization in patients with chronic coronary syndrome (CCS) have evolved from the study of cardiovascular risk factors, anatomic coronary artery disease (CAD) based modalities, and non-invasive and invasive functional ischemia testing to identify those who may most benefit from revascularization to placebo-controlled studies to assess the anti-anginal effects of revascularization.

Although revascularization has been conventionally used to manage angina, its placebo-controlled efficacy has only been conclusively tested in recent years in the ORBITA trials. The first ORBITA trial found no significant difference in exercise time or symptoms with PCI compared to a placebo procedure, highlighting the potential influence of the placebo effect in the overall observed treatment effect of PCI.^1^ However, this study was criticized for not reflecting real-world practice, as all patients underwent a six-week intensive medical optimization phase, resulting in patients taking an average of 3 antianginal agents before randomization. The subsequent trial, ORBITA-2, addressed this criticism by eliminating the medical optimization phase and taking patients off antianginal medication to test the unattenuated placebo-controlled efficacy of PCI. It demonstrated that in a placebo-controlled setting, PCI significantly improved angina symptom scores in patients not receiving anti-anginal medications.^2^ Although these trials have enhanced our understanding of the impact of revascularization on angina, some uncertainties relating to the long-term efficacy of revascularization in all-comer real-world patients remain. Furthermore, data regarding the relative efficacy of different revascularization strategies on angina are limited.

We conducted a nationwide study to evaluate the impact of revascularization with CABG and PCI on angina. We used dispensed prescriptions of long-acting nitrates as a proxy for the presence of symptoms. As a secondary objective, we compared long-acting nitrates across different revascularization strategies to assess their relative anti-anginal efficacy.

## Methods

### Data source

This is a cohort study using prospectively collected data from the Swedish Coronary Angiography and Angioplasty Registry (SCAAR) and the Swedish National Prescribed Drugs Registry.^3^ SCAAR is a nationwide registry containing data from all patients undergoing coronary angiography across 29 hospitals in Sweden with a catheterization laboratory. SCAAR is a quality registry; all patients are informed about their inclusion and provided the option to opt out. SCAAR was merged with the Swedish National Prescribed Drugs Registry to access data on medical therapies. The Swedish National Prescribed Drugs Registry contains data on all dispensed medical therapies in Sweden since 2005. SCAAR was also merged with the Swedish Cardiac Surgery Registry to collect procedural variables of CABG surgery. The study was approved by the Swedish Ethical Review Authority (Dnr 2023-00201-01) and followed appropriate ethical guidelines.

### Study population

**Fig. 1:**
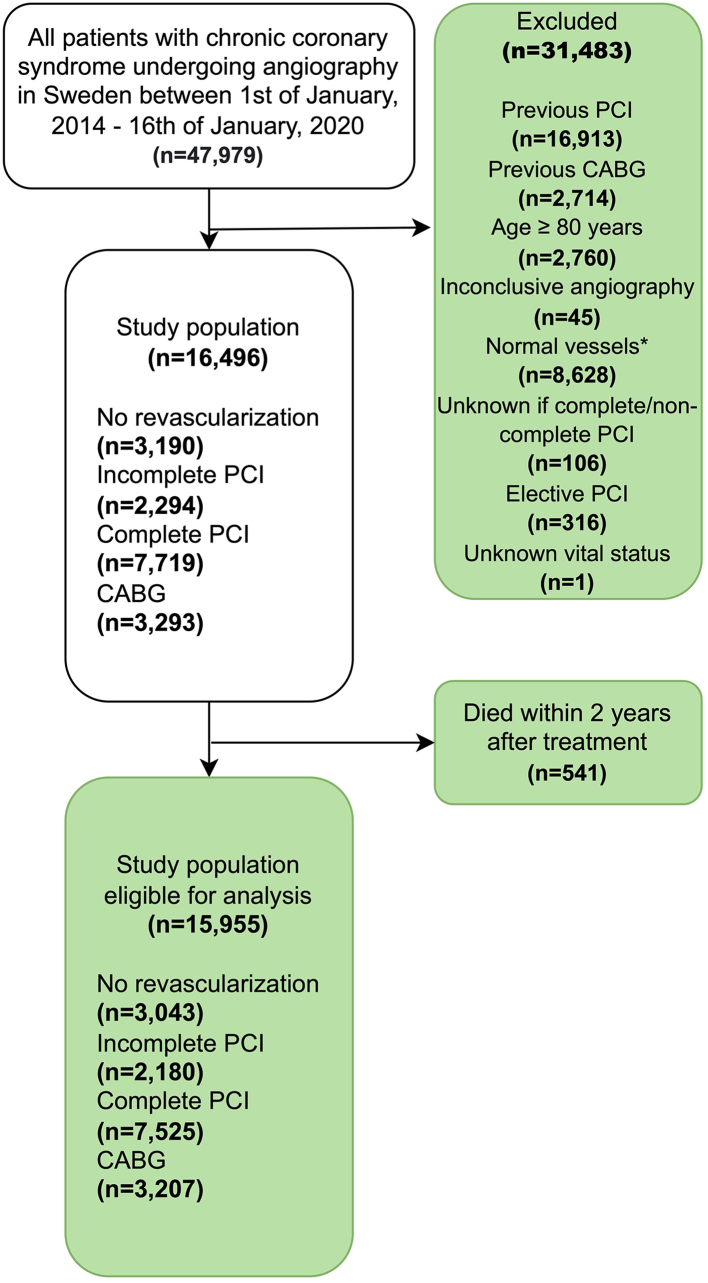
**Flowchart**, flowchart illustrating inclusion and exclusion criteria. The final study population eligible for the analysis consisted of 15,955 patients. ∗No obstructive CAD on angiography, defined as a lack of lesions with ≥50% luminal stenosis (regarded as normal vessels) CABG, coronary artery bypass graft surgery; CAD, coronary artery disease; PCI, percutaneous coronary intervention.

SCAAR was used to identify all patients with CCS admitted to coronary angiography in Sweden between 1st of January 2014 and 16th of January 2020. The inclusion date was defined as the date the patient underwent coronary angiography. A flowchart illustrating inclusion and exclusion criteria is presented in Fig. 1. Key exclusion criteria comprised previous PCI/CABG, age ≥80 years, or no obstructive CAD on angiography, defined as a lack of lesions with ≥50% luminal stenosis (regarded as normal vessels). Four groups were determined according to treatment strategy: CABG, complete revascularization with PCI, incomplete revascularization with PCI, and no revascularization. Patients undergoing ad-hoc PCI were divided into the incomplete and complete revascularization groups, respectively. Completeness of PCI was determined by the operator and permitted <10% of estimated left ventricular mass to be supplied by vessels with mild stenoses (<60%) being regarded as complete.^4^ The CABG group was defined as patients referred for CABG within 180 days after angiography. Patients not undergoing ad-hoc PCI or CABG within 180 days and admitted for elective PCI within 180 days were excluded. Only patients not undergoing ad-hoc PCI or CABG/PCI within 180 days were included in the no revascularization group.

### Study design and outcome

For the primary objective, we employed a case-crossover study design where each patient served as their own control. This study design inherently controls time-invariant confounding. In a case-crossover study, one control period and one case period are assigned to each patient. For this study, the time 1 year before the inclusion date served as the control period, and 1–2 years after the inclusion date was defined as the case period. Patients dying within 2 years after inclusion were not eligible for the analysis. We conducted an analysis investigating the proportion of patients dying within 2 years, this proportion of patients was low for all groups (541/16,496 [3.3%] for all patients) (Supplementary Figure S1). The primary outcome of this study was the use of long-acting nitrates. The use of long-acting nitrates was investigated for each period and defined as at least one dispensed drug prescription with Anatomical Therapeutic Chemical code C01DA14. Data on the use of long-acting nitrates was stored as a binary variable: 0 = no dispensed long-acting nitrates during the period, and 1 = dispensed long-acting nitrates during the period. In addition to long-acting nitrates, data was collected on dispensed statins, other lipid-lowering agents, beta-blockers, aspirin, angiotensin-converting enzyme inhibitors/angiotensin receptor blockers, calcium channel blockers, and P2Y12 inhibitors. These variables were also stored as binary variables. The anti-anginal medications Nicorandil, Trimetazidine and Ranolazine are not registered for use in Sweden and Ivabradine is uncommonly used in Sweden.

### Statistical analysis

Normally distributed continuous variables are presented as means with standard deviation. Categorical data are presented as counts with percentages. The use of long-acting nitrates was compared between the case and control period using univariable conditional Poisson regression. Results from the conditional Poisson regressions are presented as risk-ratio (RR) along with 95% confidence interval (CI) and p-value. Subgroup analyses were carried out for sex (SCAAR records sex as male and female, but does not collect information on gender identity), Canadian Cardiovascular Society Score (CCSS) grading at baseline (I vs. II-IV), disease extent on angiography (single vessel disease vs. multivessel disease), diabetes mellitus, and heart failure. An analysis was also conducted to compare the four groups using univariable and multivariable Poisson regression. For the adjusted analysis, we included the following variables: sex, age, diabetes mellitus, hypertension, inclusion year, disease extent on angiography, and CCSS at baseline. The variables used in the multivariable Poisson regression were determined a priori based on previous experience. Nine sensitivity analyses were conducted. The first was an analysis that stratified patients on the use or lack of long-acting nitrates during the control period. Second, an analysis comprising only patients with long-acting nitrates use both during the case- and control period. In this analysis, we examined whether the total dosage of long-acting nitrates, measured in milligrams, was higher or lower during the case period. Third, an analysis investigating the use of long-acting nitrates within a 90-day interval. In this analysis, the control period was 90–1 day before angiography and the case period was 360–449 after angiography. Fourth, we applied the E-value methodology in a sensitivity analysis to determine the influence of unmeasured confounding.^5^ Fifth, we conducted a sensitivity analysis for the CABG group using the surgery date as the inclusion time instead of the date of angiography. Sixth, we conducted an analysis looking at clinical outcome measures. In this descriptive analysis we assessed major adverse cardiovascular events, defined as the composite of cardiovascular death, myocardial infarction and stroke as well as its components using Kaplan–Meier plots. Seventh, we assessed the use of long-acting nitrates using negative binomial with the same fixed estimates as the conditional Poisson regression. Eighth, we performed 1:1 propensity score matching using a calliper approach with a no replacement method of the CABG and complete PCI group. The following variables were used to calculate propensity score: sex, age, diabetes mellitus, hypertension, inclusion year, disease extent on angiography, and CCSS at baseline. Following the propensity score matching, we assessed the primary outcome using conditional Poisson regression. Ninth, an analysis including only patients admitted to university hospitals was carried out. A p-value less than 0.05 was considered statistically significant. Data management and all statistical analysis were conducted in STATA (Version 18; StataCorp, Texas, LLC).

### Role of the funding source

The sponsors were not involved in the study design, collection of data, analysis of data, interpretation of data, writing of the manuscript, approving the manuscript or in the decision to submit manuscript for publication. The decision to submit manuscript was solely the authors.

## Results

### Study population

For this study, 15,955 patents with CCS were eligible (Fig. 1). Among the included patients, 3207/15,955 (20.1%) underwent CABG, 7525/15,955 (47.2%) had complete revascularizations with PCI, 2180/15,955 (13.7%) had incomplete revascularization with PCI, and 3043/15,955 (19.1%) had no revascularization. The mean age was 66.2 years (SD 8.5), and 12,403/15,955 (77.7%) of the patients were male (Table 1). The CABG group had the largest proportion of patients with three-vessel disease and/or left main disease 2704/3207 (84.3%). Patients in the complete revascularization with PCI group had the lowest mean age, 65.4 years (SD 8.7). The incomplete revascularization with PCI group had the largest proportion of patients with CCSS III-IV at baseline 752/2174 (34.6%). The no-revascularization group had the largest proportion of females 778/3043 (25.6%). Among the patients included in the CABG group, 2674/2776 (96.3%) involved left internal mammary artery and a mean of 4.6 (SD 1.3) anastomosis were used (Supplementary Table S1).

**Table 1:** Baseline characteristics.

	No revascularization	Incomplete revascularization with PCI	Complete revascularization with PCI	CABG	Missing n (%)
	(n = 3043)	(n = 2180)	(n = 7525)	(n = 3207)	
Use of long-acting nitrates 1-year prior to angiography	864/3043 (28.4%)	601/2180 (27.6%)	1676/7525 (22.3%)	989/3207 (30.8%)	0/15,955 (0.0)
Inclusion year					
2014–2016	1393/3043 (45.8%)	1141/2180 (52.3%)	3802/7525 (50.5%)	1705/3207 (53.2%)	0/15,955 (0.0)
2017–2018	1077/3043 (35.4%)	694/2180 (31.8%)	2394/7525 (31.8%)	1026/3207 (32.0%)	
2019–2020	573/3043 (18.8%)	345/2180 (15.8%)	1329/7525 (17.7%)	476/3207 (14.8%)	
Sex					
Male	2265/3043 (74.4%)	1736/2180 (79.6%)	5678/7525 (75.5%)	2724/3207 (84.9%)	0/15,955 (0.0)
Female	778/3043 (25.6%)	444/2180 (20.4%)	1847/7525 (24.5%)	483/3207 (15.1%)	
Age, mean (SD)	66.9 (8.5)	66.9 (8.4)	65.4 (8.7)	67.1 (7.8)	0/15,955 (0.0)
BMI, mean (SD)	27.8 (4.5)	27.8 (4.3)	27.6 (5.6)	27.9 (5.0)	2310/15,955 (14.5)
CCSS					
I	619/3036 (20.4%)	224/2174 (10.3%)	879/7512 (11.7%)	342/3201 (10.7%)	32/15,955 (0.2)
II	1703/3036 (56.1%)	1196/2174 (55.0%)	4464/7512 (59.4%)	1886/3201 (58.9%)	
III	667/3036 (22.0%)	740/2174 (34.0%)	2120/7512 (28.2%)	956/3201 (29.9%)	
IV	47/3036 (1.5%)	14/2174 (0.6%)	49/7512 (0.7%)	17/3201 (0.5%)	
Smoking status					
Non-smoker	1218/2982 (40.8%)	891/2136 (41.7%)	3366/7383 (45.6%)	1316/3162 (41.6%)	292/15,955 (1.8)
Previous smoker	1389/2982 (46.6%)	1005/2136 (47.1%)	3253/7383 (44.1%)	1539/3162 (48.7%)	
Smoker	375/2982 (12.6%)	240/2136 (11.2%)	764/7383 (10.3%)	307/3162 (9.7%)	
Hypertension	2332/3043 (76.6%)	1767/2180 (81.1%)	5449/7525 (72.4%)	2486/3207 (77.5%)	0/15,955 (0.0)
Hyperlipidaemia	2100 (69.3%)	1630 (74.9%)	5173 (68.9%)	2424 (76.0%)	51/15,955 (0.3)
Diabetes mellitus	879/3043 (28.9%)	625/2180 (28.7%)	1628/7525 (21.6%)	991/3207 (30.9%)	0/15,955 (0.0)
Previous myocardial infarction	398/3043 (13.1%)	344/2180 (15.8%)	579/7525 (7.7%)	283/3207 (8.8%)	0/15,955 (0.0)
Heart failure	161/3043 (5.3%)	131/2180 (6.0%)	327/7525 (4.3%)	76/3207 (2.4%)	0/15,955 (0.0)
Previous stroke	193/3043 (6.3%)	114/2180 (5.2%)	297/7525 (3.9%)	171/3207 (5.3%)	0/15,955 (0.0)
Peripheral artery disease	138/3043 (4.5%)	113/2180 (5.2%)	214/7525 (2.8%)	113/3207 (3.5%)	0/15,955 (0.0)
Renal failure	85/3043 (2.8%)	91/2180 (4.2%)	147/7525 (2.0%)	45/3207 (1.4%)	0/15,955 (0.0)
COPD	152/3043 (5.0%)	98/2180 (4.5%)	267/7525 (3.5%)	59/3207 (1.8%)	0/15,955 (0.0)
Cancer	104/3043 (3.4%)	77/2180 (3.5%)	207/7525 (2.8%)	85/3207 (2.7%)	0/15,955 (0.0)
Disease extent on angiography					
1 VD	1340/3043 (44.0%)	232/2180 (10.6%)	4876/7525 (64.8%)	105/3207 (3.3%)	0/15,955 (0.0)
2 VD	572/3043 (18.8%)	1123/2180 (51.5%)	1871/7525 (24.9%)	398/3207 (12.4%)	
3 VD and/or LM disease	1131/3043 (37.2%)	825/2180 (37.8%)	778/7525 (10.3%)	2704/3207 (84.3%)	
Use of FFR/IFR	1079/3043 (35.5%)	513/2180 (23.5%)	2039/7525 (27.1%)	588/3207 (18.3%)	0/15,955 (0.0)

BMI, body mass index; CABG, coronary artery by-pass graft surgery; CCSS, Canadian Cardiovascular Society Score grading; COPD, chronic obstructive pulmonary disease; FFR/IFR, fractional flow reserve/instantaneous wave free ratio; LM, left main; PCI, percutaneous coronary intervention; VD, vessel disease.

### Case crossover comparison

**Fig. 2:**
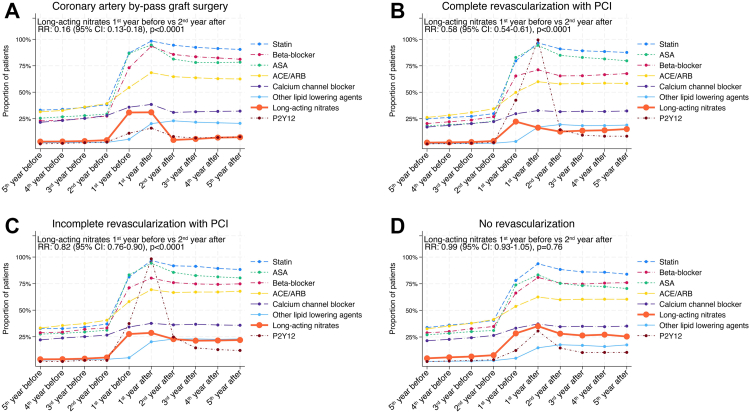
**Outcome,** Change in medical therapies before and after inclusion angiography for patients undergoing (A) coronary artery by-pass graft surgery, (B) complete revascularization with PCI, (C) incomplete revascularization with PCI, and (D) no revascularization. Use of long-acting nitrates was compared between the periods 1 year to angiography and 1–2 year after angiography and was assessed using univariable conditional Poisson regression. Results are presented as risk-ratios (RR) along with 95% confidence interval (CI) together with p-value. ACE, angiotensin converting enzyme inhibitor; ARB, angiotensin receptor blocker; ASA, acetylsalicylic acid; CI, confidence interval; PCI, percutaneous coronary intervention; RR, risk-ratio.

Revascularization was associated with a decreased use of long-acting nitrates for CABG (from 989/3207 [30.8%] to 156/3207 [4.9%]; RR: 0.16 [95% CI: 0.13–0.19], p-value <0.0001), complete revascularization with PCI (from 1676/7525 [22.3%] to 966/7525 [12.8%]; RR: 0.58 [95% CI: 0.53–0.62], p-value <0.0001) and incomplete revascularization with PCI (from 601/2180 [27.6%] to 495/2180 [22.7%]; RR: 0.82 [95% CI: 0.73–0.93], p-value = 0.0014) (Fig. 2 and Supplementary Table S2). No difference in long-acting nitrates use was observed for no revascularization (from 864/3043 [28.4%] to 856/3043 [28.1%]; RR: 0.99 [95% CI: 0.90–1.09], p-value = 0.85).

### Comparison between the four groups

An adjusted Poisson regression analysis was conducted to compare the four groups (Table 2). CABG was associated with the largest reduction in the use of long-acting nitrates, followed by complete revascularization with PCI.

**Table 2:** Comparison between the groups.

	Reference: no revascularization	Reference: incomplete revascularization	Reference: complete revascularization	Reference: CABG
**Unadjusted RR (95% CI)**				
No revascularization	Ref	1.05 (0.99–1.11), p = 0.097	1.10 (1.06–1.15), p < 0.0001	1.35 (1.28–1.42), p < 0.0001
Incomplete revascularization	0.95 (0.90–1.01), p = 0.097	Ref	1.05 (1.00–1.10), p = 0.050	1.29 (1.21–1.36), p < 0.0001
Complete revascularization	0.91 (0.87–0.95), p < 0.0001	0.95 (0.91–1.00), p = 0.050	Ref	1.22 (1.17–1.28), p < 0.0001
CABG	0.74 (0.70–0.78), p < 0.0001	0.78 (0.73–0.83), p < 0.0001	0.82 (0.78–0.86), p < 0.0001	Ref
**Adjusted RR (95% CI)**				
No revascularization	Ref	1.04 (0.98–1.10), p = 0.19	1.11 (1.07–1.17), p < 0.0001	1.30 (1.22–1.37), p < 0.0001
Incomplete revascularization	0.96 (0.91–1.02), p = 0.19	Ref	1.07 (1.02–1.13), p = 0.012	1.25 (1.17–1.33), p < 0.0001
Complete revascularization	0.90 (0.86–0.94), p < 0.0001	0.93 (0.88–0.99), p = 0.012	Ref	1.16 (1.10–1.23), p < 0.0001
CABG	0.77 (0.73–0.82), p < 0.0001	0.80 (0.75–0.85), p < 0.0001	0.86 (0.81–0.91), p < 0.0001	Ref

The four groups were analysed on long-acting nitrates use as outcome. Outcome was assessed using Poisson regression and results are presented as risk-ratios (RR) along with 95% confidence interval (CI). The adjusted multivariable Poisson regression model included sex, age, diabetes mellitus, hypertension, inclusion year, disease extent on angiography and Canadian Cardiovascular Society Score grading at baseline.

CABG, coronary artery by-pass graft surgery; CI, confidence interval; PCI, percutaneous coronary intervention; RR, risk-ratio.

### Subgroup and sensitivity analyses

**Fig. 3:**
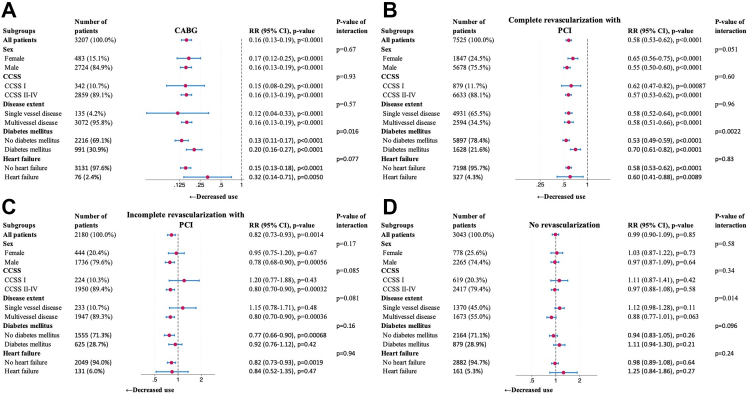
**Subgroup analysis**, Subgroup analyses were conducted for patients undergoing (A) CABG, (B) complete revascularization with PCI, (C) incomplete revascularization with PCI, and (D) no revascularization. Subgroup analyses were carried out for sex, Canadian Cardiovascular Society grading at baseline (I vs II-IV), disease extent on angiography (single vessel disease vs multi-vessel disease), diabetes mellitus and heart failure. Each subgroup was analysed on long-acting nitrates use between the control period and the case period. Outcome was assessed using conditional Poisson regression and results are presented as risk-ratios (RR) along with 95% confidence interval (CI) together with p-value of interaction. CABG, coronary artery bypass graft surgery; CCSS, Canadian Cardiovascular Society Score grading; PCI, percutaneous coronary intervention.

No statistically significant subgroup interaction was observed for the incomplete revascularization with PCI group (Fig. 3). For the CABG group and the complete revascularization with PCI group, patients without diabetes mellitus had a reduction in use of long-acting nitrates compared to patients with diabetes mellitus (p-value of interaction = 0.016, and p-value of interaction = 0.0022, respectively). For the no-revascularization group, patients with multivessel disease had a reduction in use of long-acting nitrates compared to patients with the single-vessel disease (p-value of interaction = 0.016).

**Fig. 4:**
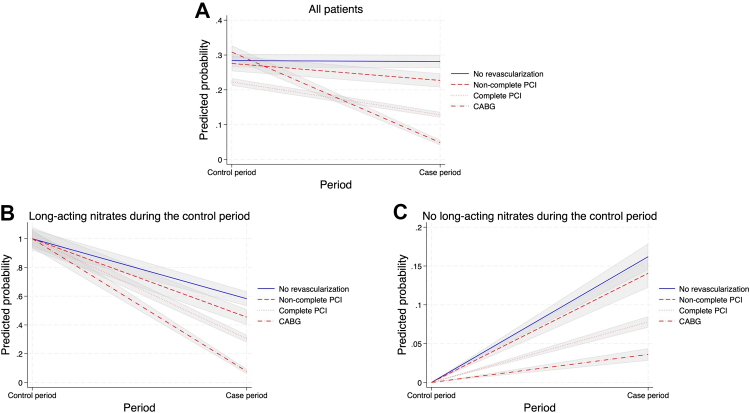
**Predicted probability,** predicted probability of a patient having long-acting nitrates before and after the index angiography was calculated using unadjusted Poison regression. The analysis was conducted on (A) all patients, (B) patients with long-acting nitrates during the control period, and (C) patients with no long-acting nitrates during the control period. CABG, coronary artery bypass graft surgery; PCI, percutaneous coronary intervention.

A sensitivity analysis was conducted by stratifying patients by use of long-acting nitrates during the control period (Fig. 4 and Supplementary Table S3). For patients without use of long-acting nitrates during the control period, CABG and complete revascularization were associated with lower use of long-acting nitrates during the case period compared to their use in the no revascularization group (adjusted RR: 0.90 [95% CI: 0.85–0.96], p-value = 0.0011), and (adjusted RR: 0.92 [95% CI: 0.87–0.96], p-value = 0.00058), respectively. For patients using long-acting nitrates during the control period, CABG, complete revascularization with PCI and incomplete revascularization with PCI were associated with lower use of long-acting nitrates during the case period compared to their use in the no revascularization group (adjusted RR: 0.16 [95% CI: 0.12–0.20], p-value <0.0001), (adjusted RR: 0.51 [95% CI: 0.45–0.58], p-value <0.0001), and (adjusted RR: 0.77 [95% CI: 0.66–0.90], p-value = 0.0013), respectively. A second sensitivity analysis included only patients with long-acting nitrates used during the case and control period (Supplementary Figure S2). This analysis showed a lower use of dispensed long-acting nitrates after CABG and complete revascularization with PCI compared to no revascularization (adjusted RR: 2.15 [95% CI: 1.24–3.72], p-value = 0.0062), and (adjusted RR: 1.45 [95% CI: 1.08–1.96], p-value = 0.015), respectively. A third sensitivity analysis was conducted comparing the use of long-acting nitrates 90–1 day before angiography with 360–449 days after angiography (Supplementary Figure S3). The results of this analysis illustrate that breaking the time points into several periods were in line with the main results. The e-values for the point estimates were 1.74 for incomplete PCI, 2.84 for complete PCI, and 11.98 for CABG, indicating that a confounder would need to be associated with both the exposure and the outcome by a RR of 1.74, 2.84, or 11.98 each, to explain away the observed association (Supplementary Table S4). A sensitivity analysis was conducted for the CABG group by using the time of surgery as the inclusion date (Supplementary Figure S4). The results of this analysis were in-line with the main results for the CABG group. In the supplementary analysis investigating clinical outcomes, the 2-year Kaplan–Meier estimate for major adverse cardiovascular events was 7.0% for CABG, 4.8% for complete revascularization with PCI, 10.2% for incomplete revascularization with PCI and 6.8% for no revascularization (Supplementary Figure S5). In a sensitivity analysis using negative binomial to assess the primary outcome, the results were consistent with the conditional Poisson model (Supplementary Table S5). In a propensity score matched cohort investigating outcome for complete PCI and the CABG group, baseline characteristics were similar between the two group (Supplementary Table S6). In this analysis, a similar reduction in long-acting nitrate use was observed for both groups compared to the complete cohort (Supplementary Figure S6). In a sensitivity analysis including only patients admitted to university hospitals (n = 5183), similar risk-ratios in use of long-acting nitrates were observed as compared with the main cohort (Supplementary Figure S7).

## Discussion

This study assessed the effectiveness of revascularization on angina symptoms by examining the use of long-acting nitrates in patients with CCS. Patients who underwent revascularization with CABG or PCI saw a reduction in of long-acting nitrates, whereas no such reduction was observed in patients who did not receive revascularization. Furthermore, CABG appears to have a more pronounced effect in reducing the use of anti-anginal medications compared to PCI. Our results highlight the real-world effectiveness of revascularization by analysing a comprehensive cohort of 15,000 all-comer patients. Despite the observational nature of this study and the limitation of retrospective data, this study provides valuable insights into the effectiveness of CABG and PCI in reducing the use of long-acting nitrates, probably reflecting reduced angina symptoms.

The proportions of patients with CCS undergoing revascularization continue to increase.^6^ Today, patients with CCS comprise one-third of all patients undergoing PCI.^6^^,^^7^ The European Society of Cardiology (ESC) endorses revascularization as an adjunct to medical therapy for patients with CCS to relieve symptoms when angina persists despite treatment with anti-anginal medications and/or to improve prognosis (Class 1, Level A).^8^ Previous research has shown that angina is significantly associated with reduced quality of life.^9^^,^^10^ This is reflected in current guidelines, which recognize the role of revascularization in improving quality of life and relieving symptoms. However, guidelines do not specifically endorse revascularization to decrease reliance on medical therapy, a question raised after the ORBITA 2 trial. The effect of revascularization on the use of anti-anginal medications has been previously documented. Although FAME 2 did not specifically report on long-acting nitrates, other anti-anginal medications were reduced during the first six months after randomization.^11^ This effect was diminished after six months. A similar trend was noted in the ISCHEMIA trial.^12^ The COURAGE trial also observed the reduced impact over time as the quality-of-life benefits of revascularization gradually diminished.^13^ In contrast to these trials, the SYNTAX trial showed substantial and sustained quality of life benefits and reduced long-acting nitrates use five years after revascularization.^14^ The findings of our study align with the SYNTAX trial, demonstrating that the effects of revascularization persist over the long term. Furthermore, we observed that revascularization reduced the future use for these medications in patients not using long-acting nitrates at baseline. Together, these observations support the notion that revascularization effectively reduces angina symptoms and could further motivate its use for long-term symptom control. The subgroup analyses indicated no differences depending on sex, CCSS class, heart failure, or single versus multivessel disease among patients undergoing revascularization. In the no-revascularization group, a significant subgroup interaction was observed. Patients with multi-vessel disease had a reduction in use of long-acting nitrates compared to single-vessel disease (interaction p-value: 0.016). This finding may seem counterintuitive but is consistent with the recent study by Simader et al., which demonstrates that disease severity is poorly correlated with symptom burden.^15^ A conceivable explanation for these findings could be the fact that these patients avoid activities that may trigger angina.

Our secondary aim was to compare the use of long-acting nitrates across different revascularization strategies. Of interest, CABG was associated with the most significant reduction in long-acting nitrate use, followed by complete PCI, and incomplete PCI. Our findings thus encourage prioritizing complete revascularization for symptom relief and may inform future guidelines. While the first ORBITA trial highlighted a potential influence of a placebo effect of PCI, similar data for CABG remains scarce, and their comparative placebo effect remains unknown.^1^ While it may be tempting to speculate that the placebo effect associated with cardiac surgery could be greater than that of PCI, current evidence suggests that the placebo effect in classical surgery is generally lower in traditional surgical procedures than in percutaneous interventions.^16^ Therefore, the observed benefit of CABG is likely attributable to its genuine therapeutic effect rather than a placebo response. Several factors likely contribute to this finding. In the ISCHEMIA trial, patients with more frequent baseline angina derived a greater benefit from revascularization. The results of this study align with this finding, showing that patients already on long-acting nitrates at baseline were more likely to discontinue their use over time if they underwent revascularization than the overall population and those who did not receive revascularization. Another plausible explanation for the high efficacy of CABG is that it potentially improves overall circulation and reduce vascular aging more effectively than PCI but also results in less repeat revascularizations which may result in prolonged nitrate use. Despite the superior efficacy of CABG in reducing the use of long-acting nitrates, other important aspects such as lesion anatomy, patients’ comorbidities and upfront risk/cost needs to be considered in the selection of treatment strategy.

The ORBITA-STAR trial demonstrated that comparing patient symptoms before angiography with those experienced during low-pressure balloon occlusion of coronary stenosis provided a strong predictor of symptom improvement following PCI, based on similarity scores.^17^ Although this procedure is relatively straightforward during PCI, an even simpler approach could involve initiating treatment with long-acting nitrates before referring patients for coronary angiography. Patients who experience symptom relief from nitrates might be more likely to benefit from revascularization. Conversely, having information on patients whose symptoms do not improve after initiation of long-acting nitrates is valuable, as it allows clinicians to correlate symptom severity with angiographic findings, helping to identify those who may not have angina and guiding more precise treatment decisions.

This study has several limitations. The primary limitation is the use of long-acting nitrate prescriptions as a surrogate for angina symptoms. Consequently, we rely on the use of long-acting nitrates as an indirect indicator of symptoms as symptom diaries and quality-of-life metrics were not available. Unfortunately, the National Prescribed Drug Registry does not capture the reasons for medication discontinuation (e.g., side effects vs symptom resolution), limiting our ability to establish a direct causal link. While dispensed prescriptions might not fully reflect actual usage, we believe it is the most reliable method to study medication use. However, there are other causes for not using long-acting nitrates such as adverse events. Another possible reason for discontinuing long-acting nitrates is prescription bias as it is reasonable to believe doctors and surgeons are more likely to discontinue long-acting nitrates after an intervention. However, as the results of this study demonstrate that the effects of revascularization on dispensed long-acting nitrates persist over 5-years, we believe that prescription bias cannot explain away the observed reduction of long-acting nitrates. If prescription bias was the case, we would expect to see a later increase on dispensed prescriptions. The employed case-crossover design effectively addresses our primary research question and minimizes confounding. However, evaluating our secondary objectives presents several challenges due to the observational nature of the study. Variations in baseline characteristics among the four groups, along with the non-randomized nature of the treatment strategies, may have introduced treatment bias. Patients with the most severe CAD and angina—those likely to benefit the most from revascularization—were more often treated with CABG or complete revascularization using PCI. In contrast, patients with milder or atypical symptoms may have been more frequently subjected to incomplete revascularization or no revascularization at all. The lack of symptomatic improvement after the procedure in these latter groups could explain the higher rates of continued medication use. Additionally, this group may also include patients with more diffuse CAD or underlying microvascular obstruction, which could further contribute to persistent symptoms. The CABG and the complete PCI group experience lower unadjusted rates of mortality as compared with the incomplete PCI and the no revascularization group, possibly reflected by the different patient profiles. Finally, as SCAAR include data from 29 hospitals in Sweden, inappropriateness and/or heterogeneity of decision-making among the centres may introduce bias.

Revascularization reduces the use of long-acting nitrates patients with CCS, suggesting angina symptom improvement. CABG appears to provide a more significant benefit than PCI, with complete PCI demonstrating greater effectiveness than incomplete revascularization. Furthermore, this analysis showed that revascularization, regardless of the method or extent, reduced the use of long-acting nitrates in patients who were using them at baseline.

## Contributors

DE and MAM conceptualized the study. SvK was responsible for data management, analysed the data and drafted the manuscript. SvK and MAM directly accessed and verified the underlying data and were responsible for the decision to submit the manuscript. All authors contributed to the design of the study. All authors interpreted the results, intellectually and critically revised the manuscript, and approved its final form.

## Data sharing statement

Due to patient secrecy laws, data are not publicly available, interested researchers can contact us and we may help with presenting aggregated data.

## Declaration of interests

TJ received institutional grants from MSD in another project. SJ received institutional grants from Novo Nordisk, Amgen, Jansen, BMS, Medtronic and Edwards. SJ received payment for lectures from Medtronic. SvK, TS, RK, EO, SZ, JS, DE and MAM declare no support for the submitted work from any organization, no financial relationship in the past three years with any organization that might have an interest in the submitted work, and no other relationships or activities that could appear to have influenced the submitted work.
