# Supplementary material for: Long-acting nitrate use before and after revascularization to evaluate angina in chronic coronary syndrome: a case-crossover study from SCAAR
Source: Lancet Reg Health Eur. 2025 Oct 28;60:101507. doi: 10.1016/j.lanepe.2025.101507 (PMC12596951; doi:10.1016/j.lanepe.2025.101507)
Supplement: Supplementary Materials [file mmc1.docx]

Supplementary materials

**Elements included in the supplementary materials:**

**Supplementary Table 1.** CABG procedural aspects

**Supplementary Table 2**. Anti-anginal medications over time

**Supplementary Table 3**. Sensitivity analysis stratifying patients on long-acting nitrates use at the control period

**Supplementary Table 4**. E-value

**Supplementary Table 5.** Negative binomial sensitivity analysis

**Supplementary Table 6.** Propensity score matched cohort of CABG and complete PCI

**Supplementary Figure 1**. All-cause mortality

**Supplementary Figure 2**. Sensitivity analysis on patients with long-acting nitrates both during the case and control period

**Supplementary Figure 3**. Sensitivity analysis on patients with long-acting nitrates both during the case and control period

**Supplementary Figure 4**. CABG surgery time sensitivity analysis

**Supplementary Figure 5.** Clinical outcomes

**Supplementary Figure 6**. Propensity score matched analysis of complete PCI and CABG

**Supplementary Figure 7.** Sensitivity analysis including only patients admitted to university hospitals

**Supplementary Table 1. CABG procedural aspects**

|  |  | 1 vessel disease | 2 vessel disease | 3 vessel disease and/or left main |
| --- | --- | --- | --- | --- |
|  |  | (n=105) | (n=398) | (n=2704) |
| Left internal mammary artery |  | 81/91 (89.0%) | 316/327 (96.6%) | 2277/2358 (96.6%) |
| Right internal mammary artery |  | 3/91 (3.3%) | 20/327 (6.1%) | 192/2358 (8.1%) |
| Number of anastomoses used | 1 | 30/90 (33.3%) | 5/326 (1.5%) | 7/2344 (0.3%) |
|  | 2 | 17/90 (18.9%) | 8/326 (2.5%) | 20/2344 (0.9%) |
|  | 3 | 29/90 (32.2%) | 123/326 (37.7%) | 318/2344 (13.6%) |
|  | 4 | 10/90 (11.1%) | 102/326 (31.3%) | 638/2344 (27.2%) |
|  | 5 | 2/90 (2.2%) | 64/326 (19.6%) | 707/2344 (30.2%) |
|  | 6 | 2/90 (2.2%) | 22/326 (6.7%) | 474/2344 (20.2%) |
|  | 7 | 0/90 (0.0%) | 2/326 (0.6%) | 145/2344 (6.2%) |
|  | 8 | 0/90 (0.0%) | 0/326 (0.0%) | 31/2344 (1.3%) |
|  | 9 | 0/90 (0.0%) | 0/326 (0.0%) | 2/2344 (0.1%) |
|  | 10 | 0/90 (0.0%) | 0/326 (0.0%) | 2/2344 (0.1%) |
| Number of anastomoses used, mean (SD) |  | 2.4 (1.2) | 3.9 (1.1) | 4.8 (1.2) |

CABG = coronary artery by-pass graft.

**Supplementary Table 2. Anti-anginal medications over time**

|  |  | 3^rd^ year before | 1^st^ year before | 2^nd^ year after | 4^th^ year after |
| --- | --- | --- | --- | --- | --- |
| Any anti-anginal drug | CABG | 1291/3207 (40.3%) | 2782/3207 (86.7%) | 2878/3207 (89.7%) | 1918/2182 (87.9%) |
|  | Complete PCI | 2657/7525 (35.3%) | 5909/7525 (78.5%) | 5797/7525 (77.0%) | 3845/4929 (78.0%) |
|  | Incomplete PCI | 975/2180 (44.7%) | 1788/2180 (82.0%) | 1873/2180 (85.9%) | 1200/1431 (83.9%) |
|  | No revascularization | 1371/3043 (45.1%) | 2393/3043 (78.6%) | 2605/3043 (85.6%) | 1556/1816 (85.7%) |
| Long-acting nitrates | CABG | 122/3207 (3.8%) | 989/3207 (30.8%) | 156/3207 (4.9%) | 155/2182 (7.1%) |
|  | Complete PCI | 223/7525 (3.0%) | 1676/7525 (22.3%) | 966/7525 (12.8%) | 694/4929 (14.1%) |
|  | Incomplete PCI | 95/2180 (4.4%) | 601/2180 (27.6%) | 495/2180 (22.7%) | 307/1431 (21.5%) |
|  | No revascularization | 202/3043 (6.6%) | 864/3043 (28.4%) | 856/3043 (28.1%) | 494/1816 (27.2%) |
| Beta-blockers | CABG | 812/3207 (25.3%) | 2344/3207 (73.1%) | 2749/3207 (85.7%) | 1798/2182 (82.4%) |
|  | Complete PCI | 1786/7525 (23.7%) | 4921/7525 (65.4%) | 4926/7525 (65.5%) | 3283/4929 (66.6%) |
|  | Incomplete PCI | 698/2180 (32.0%) | 1549/2180 (71.1%) | 1655/2180 (75.9%) | 1063/1431 (74.3%) |
|  | No revascularization | 998/3043 (32.8%) | 2017/3043 (66.3%) | 2297/3043 (75.5%) | 1373/1816 (75.6%) |
| Calcium channel blockers | CABG | 810/3207 (25.3%) | 1150/3207 (35.9%) | 992/3207 (30.9%) | 696/2182 (31.9%) |
|  | Complete PCI | 1532/7525 (20.4%) | 2237/7525 (29.7%) | 2380/7525 (31.6%) | 1569/4929 (31.8%) |
|  | Incomplete PCI | 546/2180 (25.0%) | 745/2180 (34.2%) | 788/2180 (36.1%) | 516/1431 (36.1%) |
|  | No revascularization | 740/3043 (24.3%) | 1012/3043 (33.3%) | 1054/3043 (34.6%) | 629/1816 (34.6%) |

CABG = coronary artery bypass graft surgery; PCI = percutaneous coronary intervention.

**Supplementary Table 3. Sensitivity analysis stratifying patients on long-acting nitrates use at the control period**

| **No long-acting nitrates at the control period** | | | | |
| --- | --- | --- | --- | --- |
|  | **Unadjusted RR (95% CI)** | | | |
|  | Reference: No revascularization | Reference: Incomplete revascularization | Reference: Complete revascularization | Reference: CABG |
| No revascularization | Ref | 1.02 (0.96-1.08), p=0.55 | 1.08 (1.03-1.13), p=0.0014 | 1.12 (1.06-1.19), p<0.0001 |
| Incomplete revascularization | 0.98 (0.92-1.04), p=0.55 | Ref | 1.06 (1.00-1.11), p=0.035 | 1.10 (1.04-1.17), p=0.0023 |
| Complete revascularization | 0.93 (0.89-0.97), p=0.0014 | 0.95 (0.90-1.00), p=0.035 | Ref | 1.04 (0.99-1.09), p=0.10 |
| CABG | 0.89 (0.84-0.94), p<0.0001 | 0.91 (0.85-0.97), p=0.0023 | 0.96 (0.92-1.01), p=0.10 | Ref |
|  | **Adjusted RR (95% CI)** | | | |
|  | Reference: No revascularization | Reference: Incomplete revascularization | Reference: Complete revascularization | Reference: CABG |
| No revascularization | Ref | 1.03 (0.96-1.09), p=0.40 | 1.09 (1.04-1.14), p=0.00058 | 1.11 (1.04-1.18), p=0.0011 |
| Incomplete revascularization | 0.97 (0.91-1.04), p=0.40 | Ref | 1.06 (1.00-1.12), p=0.045 | 1.08 (1.01-1.15), p=0.026 |
| Complete revascularization | 0.92 (0.87-0.96), p=0.00058 | 0.94 (0.89-1.00), p=0.045 | Ref | 1.02 (0.96-1.08), p=0.59 |
| CABG | 0.90 (0.85-0.96), p=0.0011 | 0.93 (0.87-0.99), p=0.026 | 0.98 (0.93-1.04), p=0.59 | Ref |
| **Long-acting nitrates at the control period** | | | | |
|  | **Unadjusted RR (95% CI)** | | | |
|  | Reference: No revascularization | Reference: Incomplete revascularization | Reference: Complete revascularization | Reference: CABG |
| No revascularization | Ref | 1.28 (1.11-1.49), p=0.00096 | 1.91 (1.69-2.16), p<0.0001 | 7.58 (5.95-9.64), p<0.0001 |
| Incomplete revascularization | 0.78 (0.67-0.90), p=0.00096 | Ref | 1.49 (1.29-1.73), p<0.0001 | 5.91 (4.58-7.62), p<0.0001 |
| Complete revascularization | 0.52 (0.46-0.59), p<0.0001 | 0.67 (0.58-0.78), p<0.0001 | Ref | 3.96 (3.11-5.04), p<0.0001 |
| CABG | 0.13 (0.10-0.17), p<0.0001 | 0.17 (0.13-0.22), p<0.0001 | 0.25 (0.20-0.32), p<0.0001 | Ref |
|  | **Adjusted RR (95% CI)** | | | |
|  | Reference: No revascularization | Reference: Incomplete revascularization | Reference: Complete revascularization | Reference: CABG |
| No revascularization | Ref | 1.30 (1.11-1.53), p=0.0013 | 1.96 (1.72-2.23), p<0.0001 | 6.31 (4.89-8.14), p<0.0001 |
| Incomplete revascularization | 0.77 (0.66-0.90), p=0.0013 | Ref | 1.50 (1.28-1.77), p<0.0001 | 4.85 (3.73-6.32), p<0.0001 |
| Complete revascularization | 0.51 (0.45-0.58), p<0.0001 | 0.66 (0.56-0.78), p<0.0001 | Ref | 3.23 (2.48-4.20), p<0.0001 |
| CABG | 0.16 (0.12-0.20), p<0.0001 | 0.21 (0.16-0.27), p<0.0001 | 0.31 (0.24-0.40), p<0.0001 | Ref |

The four groups were analysed on long-acting nitrates use at the case period as outcome. Patients were stratified on long-acting nitrates use at baseline. Outcome was assessed using Poisson regression and results are presented as risk-ratios (RR) along with 95% confidence interval (CI) together with p-value of interaction. The adjusted multivariable Poisson regression model included sex, age, diabetes mellitus, hypertension, inclusion year, disease extent on angiography and Canadian Cardiovascular Society Score grading at baseline.

CABG = coronary artery bypass graft surgery; CI = confidence interval; PCI = percutaneous coronary intervention; RR = risk-ratio.

**Supplementary Table 4. E-value**

|  | **E-value for point estimate** | **E-value for upper bound of CI** |
| --- | --- | --- |
| CABG | 11.98 | 10.00 |
| Complete PCI | 2.84 | 2.61 |
| Incomplete PCI | 1.74 | 1.36 |

E-values was calculated using effect estimates from *Figure 2. Outcome*

**Supplementary Table 5. Negative binomial sensitivity analysis**

|  | **RR (95% CI)** | **P-value** |
| --- | --- | --- |
| CABG | 0.16 (0.13-0.18) | < 0.0001 |
| Complete PCI | 0.58 (0.54-0.61) | < 0.0001 |
| Incomplete PCI | 0.82 (0.76-0.90) | < 0.0001 |
| No revascularization | 0.99 (0.93-1.05) | 0.76 |

Change in medical therapies before and after inclusion angiography. Use of long-acting nitrates was compared between the periods 1 year to angiography and 1-2 year after angiography and was assessed using univariable negative binomial regression. Results are presented as risk-ratios (RR) along with 95% confidence interval (CI) together with p-value.

CABG = coronary artery by-pass graft; CI = confidence interval; PCI = percutaneous coronary intervention; RR = risk-ratio.

**Supplementary Table 6. Propensity score matched cohort of CABG and complete PCI**

|  |  | Complete revascularization with PCI | CABG |
| --- | --- | --- | --- |
|  |  | (n=1164) | (n=1164) |
| Use of long-acting nitrates 1-year prior to angiography |  |  |  |
| Inclusion year | 2014-2016 | 631/1164 (54.2%) | 626/1164 (53.8%) |
|  | 2017-2018 | 349/1164 (30.0%) | 345/1164 (29.6%) |
|  | 2019-2020 | 184/1164 (15.8%) | 193/1164 (16.6%) |
| Sex | Male | 947/1164 (81.4%) | 926/1164 (79.6%) |
|  | Female | 217/1164 (18.6%) | 238/1164 (20.4%) |
| Age, mean (SD) |  | 66.5 (8.1) | 65.8 (8.1) |
| BMI, mean (SD) |  | 27.8 (9.5) | 28.1 (6.4) |
| CCSS | I | 114/1162 (9.8%) | 133/1162 (11.4%) |
|  | II | 666/1162 (57.3%) | 655/1162 (56.4%) |
|  | III | 373/1162 (32.1%) | 363/1162 (31.2%) |
|  | IV | 9/1162 (0.8%) | 11/1162 (0.9%) |
| Smoking status | Non-smoker | 530/1143 (46.4%) | 483/1148 (42.1%) |
|  | Previous smoker | 502/1143 (43.9%) | 546/1148 (47.6%) |
|  | Smoker | 111/1143 (9.7%) | 119/1148 (10.4%) |
| Hypertension |  | 896/1164 (77.0%) | 911/1164 (78.3%) |
| Hyperlipidaemia |  | 839/1161 (72.3%) | 881/1157 (76.1%) |
| Diabetes mellitus |  | 290/1164 (24.9%) | 352/1164 (30.2%) |
| Previous myocardial infarction |  | 113/1164 (9.7%) | 89/1164 (7.6%) |
| Heart failure |  | 50/1164 (4.3%) | 28/1164 (2.4%) |
| Previous stroke |  | 68/1164 (5.8%) | 61/1164 (5.2%) |
| Peripheral artery disease |  | 40/1164 (3.4%) | 43/1164 (3.7%) |
| Renal failure |  | 22/1164 (1.9%) | 21/1164 (1.8%) |
| COPD |  | 29/1164 (2.5%) | 32/1164 (2.7%) |
| Cancer |  | 36/1164 (3.1%) | 32/1164 (2.7%) |
| Disease extent on angiography | 1 VD | 104/1164 (8.9%) | 104/1164 (8.9%) |
|  | 2 VD | 392/1164 (33.7%) | 392/1164 (33.7%) |
|  | 3 VD and/or LM disease | 668/1164 (57.4%) | 668/1164 (57.4%) |
| Use of FFR/IFR |  | 370/1164 (31.8%) | 261/1164 (22.4%) |

BMI = body mass index; CABG = coronary artery by-pass graft surgery; CCSS = Canadian Cardiovascular Society Score grading; COPD = chronic obstructive pulmonary disease; FFR/IFR = fractional flow reserve/instantaneous wave free ratio; LM = left main; PCI = percutaneous coronary intervention; VD = vessel disease.

**
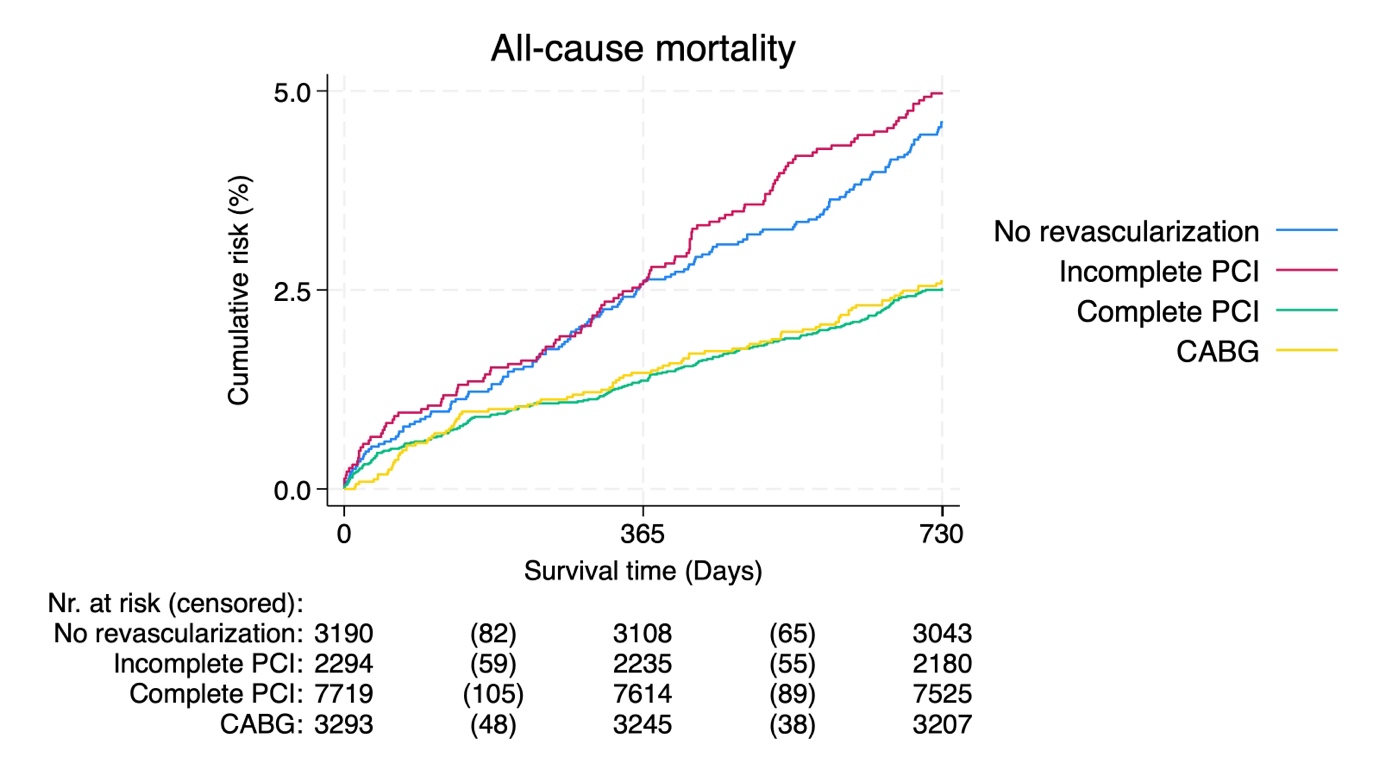
**

**Supplementary Figure 1. All-cause mortality,** Kaplan-Meier plot illustrating the event rate of all-cause mortality within the first two years after index angiography. Patients surviving after 2 years where eligible for the analysis; 3043 patients in the no revascularization group, 2180 patients in the incomplete revascularization with PCI group, 7525 patients in the complete revascularization with PCI group and 3207 patients in the CABG group.

CABG = coronary artery bypass graft surgery; PCI = percutaneous coronary intervention.


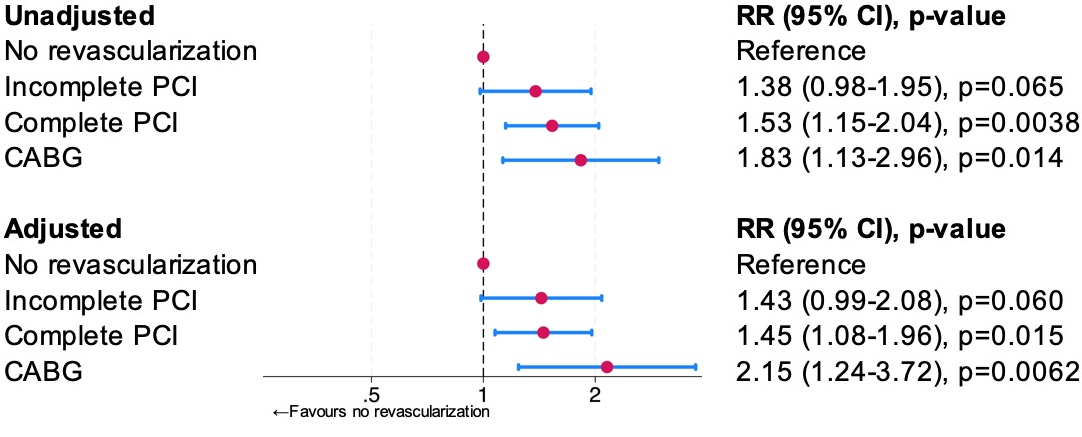


**Supplementary Figure 2. Sensitivity analysis on patients with long-acting nitrates both during the case and control period,** the four groups were analysed on total dispensed milligrams of long-acting nitrates as outcome. Only patients with long-acting nitrates use during the case and control period were included. Outcome was assessed using Poisson regression and results are presented as risk-ratios (RR) along with 95% confidence interval (CI). The adjusted multivariable Poisson regression model included sex, age, diabetes mellitus, hypertension, inclusion year, disease extent on angiography and Canadian Cardiovascular Society Score grading at baseline.

CABG = coronary artery bypass graft surgery; CI = confidence interval; RR = risk-ratio; PCI = percutaneous coronary intervention.


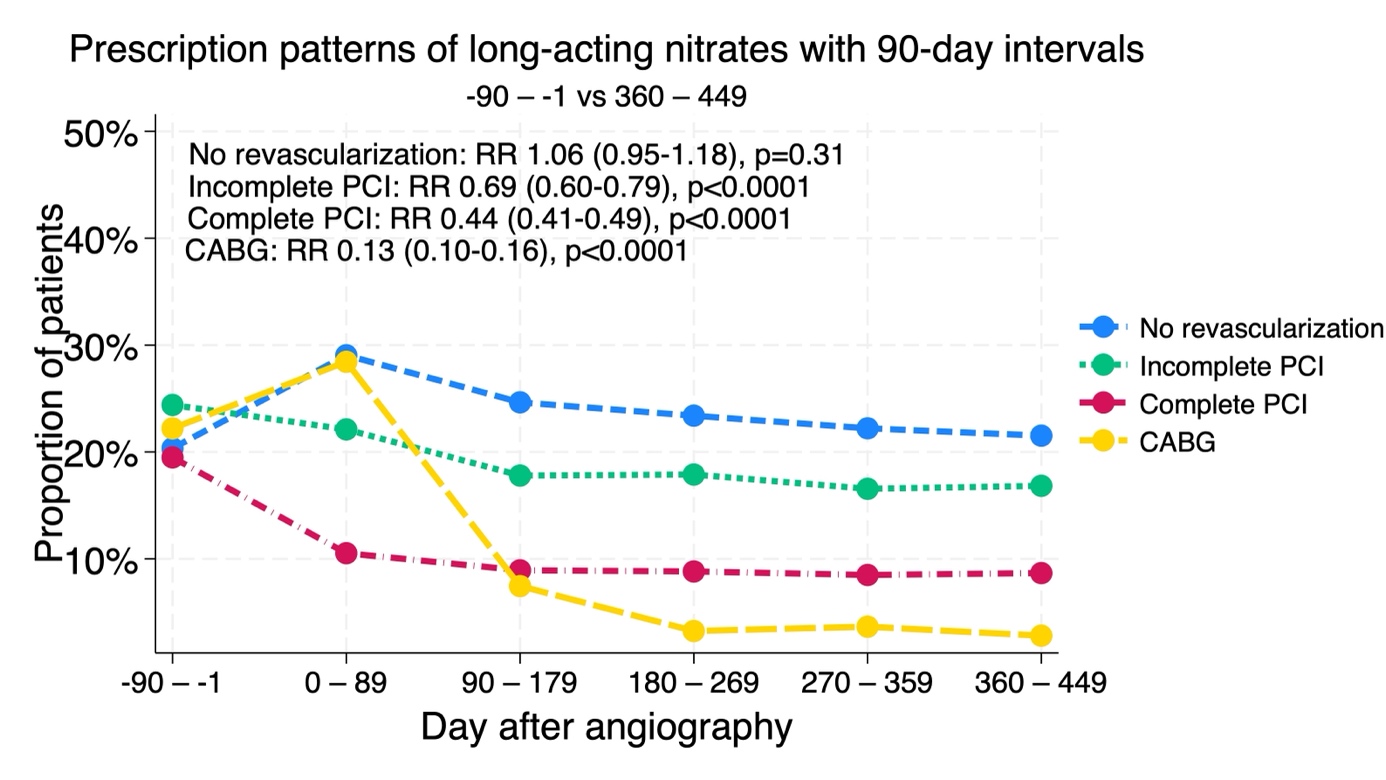


**Supplementary Figure 3. Dispensed prescriptions of long-acting nitrates with 90-day intervals,** change in medical therapies before and after inclusion angiography. Use of long-acting nitrates was compared between the periods 90 days to angiography and 360-449 days after angiography and was assessed using univariable conditional Poisson regression. Results are presented as risk-ratios (RR) along with 95% confidence interval (CI) together with p-value.

CABG = coronary artery bypass graft surgery; CI = confidence interval; RR = risk-ratio; PCI = percutaneous coronary intervention.


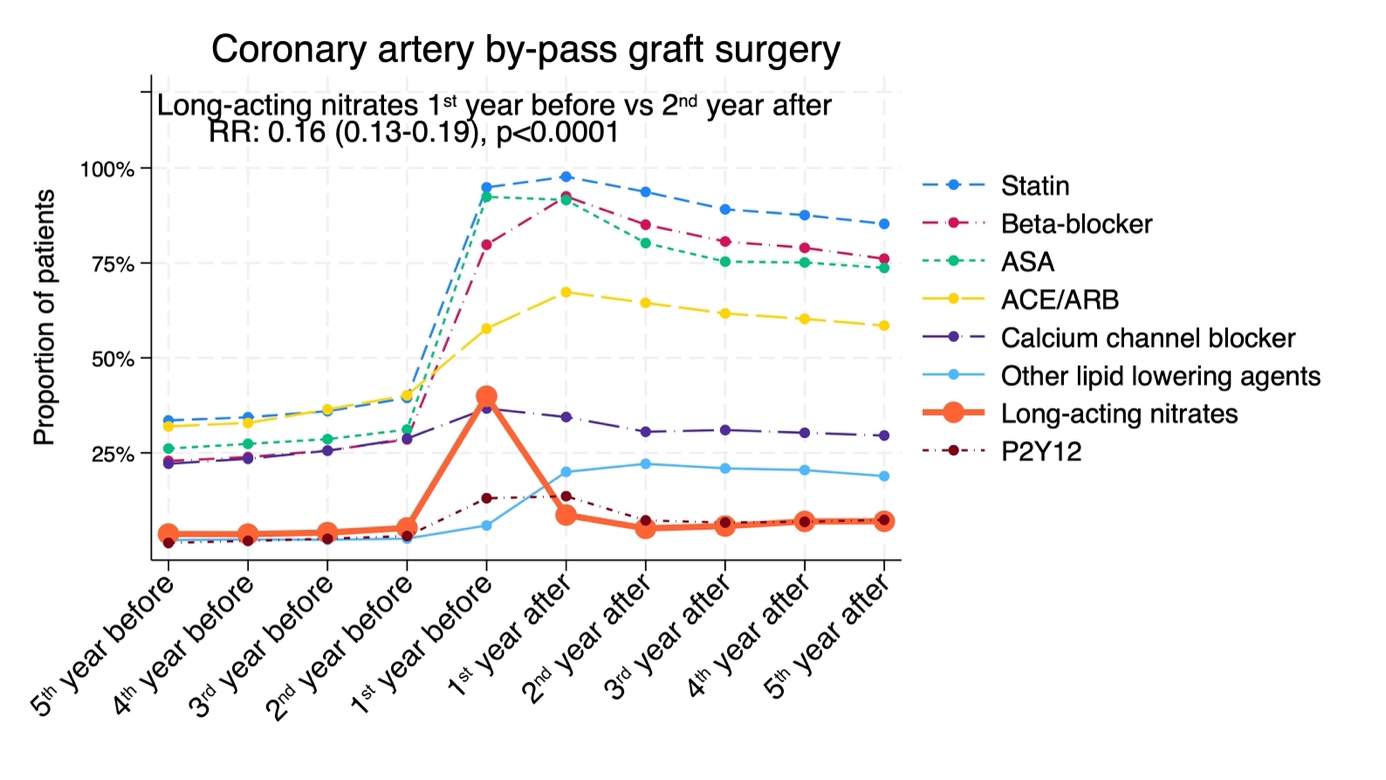


**Supplementary Figure 4. CABG surgery time sensitivity analysis,** Change in medical therapies before and after CABG surgery. Use of long-acting nitrates was compared between the periods 1 year to surgery and 1-2 year after surgery and was assessed using univariable conditional Poisson regression. Results are presented as risk-ratios (RR) along with 95% confidence interval (CI) together with p-value.

ACE = angiotensin converting enzyme inhibitor; ARB = angiotensin receptor blocker; ASA = acetylsalicylic acid; CI = confidence interval; PCI = percutaneous coronary intervention; RR = risk-ratio.


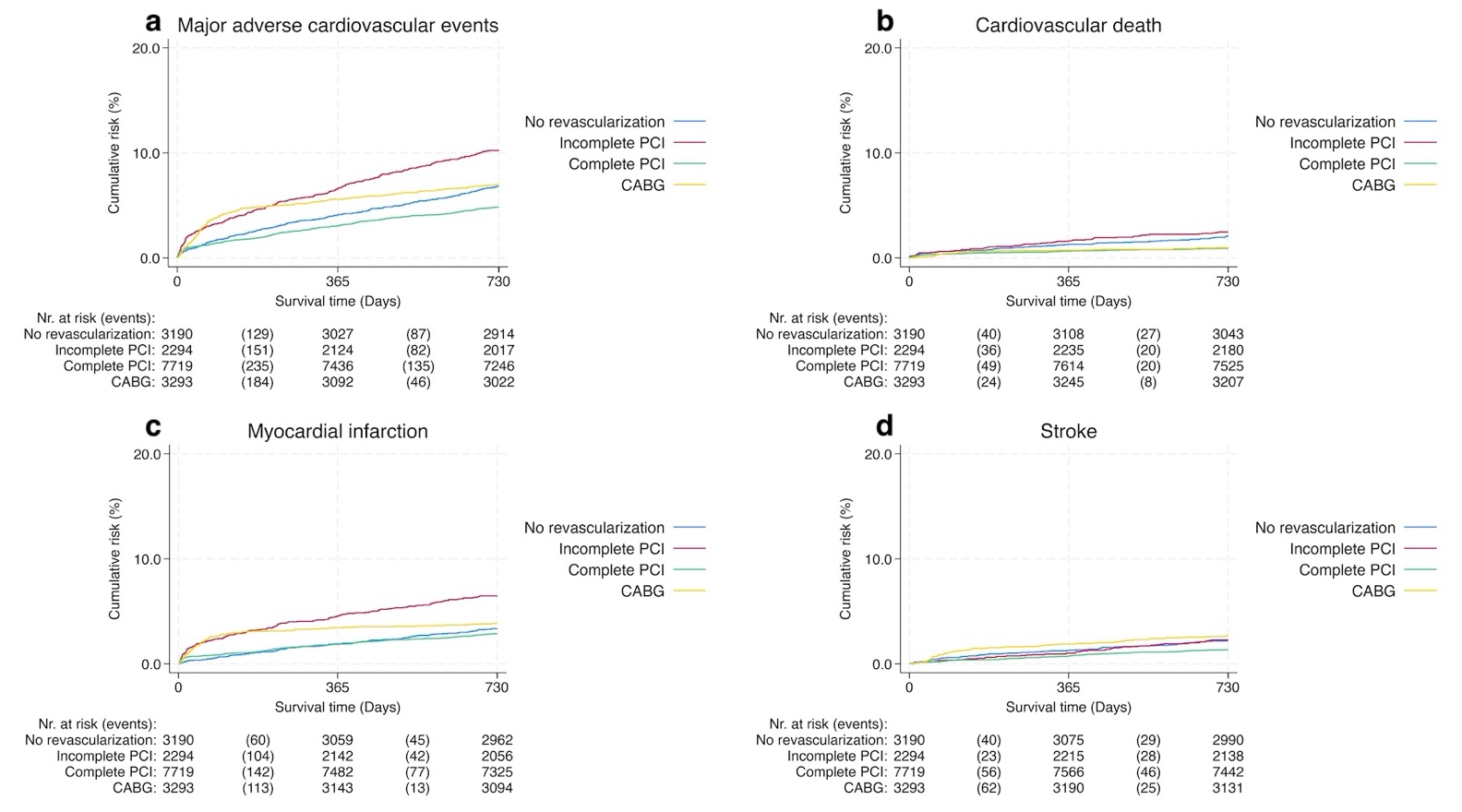


**Supplementary Figure 5. Clinical outcomes,** Kaplan-Meier plot illustrating the event rate of major adverse cardiovascular events, cardiovascular death, myocardial infarction and stroke within the first two years after index angiography. Major adverse cardiovascular events were defined as the composite of cardiovascular death, myocardial infarction and stroke.

CABG = coronary artery bypass graft surgery; PCI = percutaneous coronary intervention.


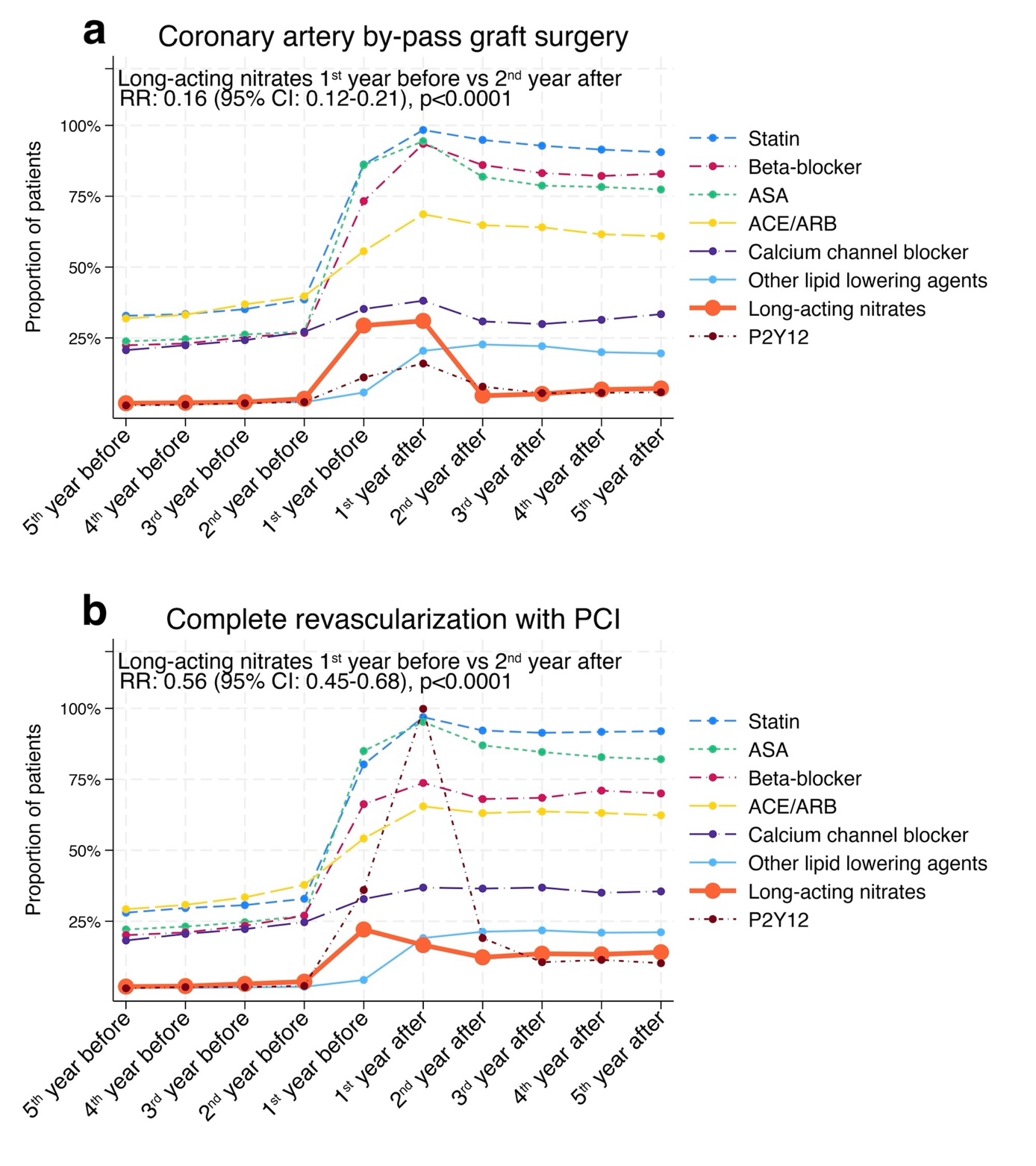


**Supplementary Figure 6. Propensity score matched analysis of complete PCI and CABG,** 1:1 propensity score matching using a calliper method with a no replacement method of the CABG and complete PCI group. The following variables were used to calculate propensity score: sex, age, diabetes mellitus, hypertension, inclusion year, disease extent on angiography, and CCSS at baseline. Following the propensity score matching, we assessed use of long-acting nitrates between the periods 1 year to angiography and 1-2 year after angiography and was assessed using univariable conditional Poisson regression. Results are presented as risk-ratios (RR) along with 95% confidence interval (CI) together with p-value.

ACE = angiotensin converting enzyme inhibitor; ARB = angiotensin receptor blocker; ASA = acetylsalicylic acid; CCSS = Canadian Cardiovascular Society Score; CI = confidence interval; PCI = percutaneous coronary intervention; RR = risk-ratio.

**
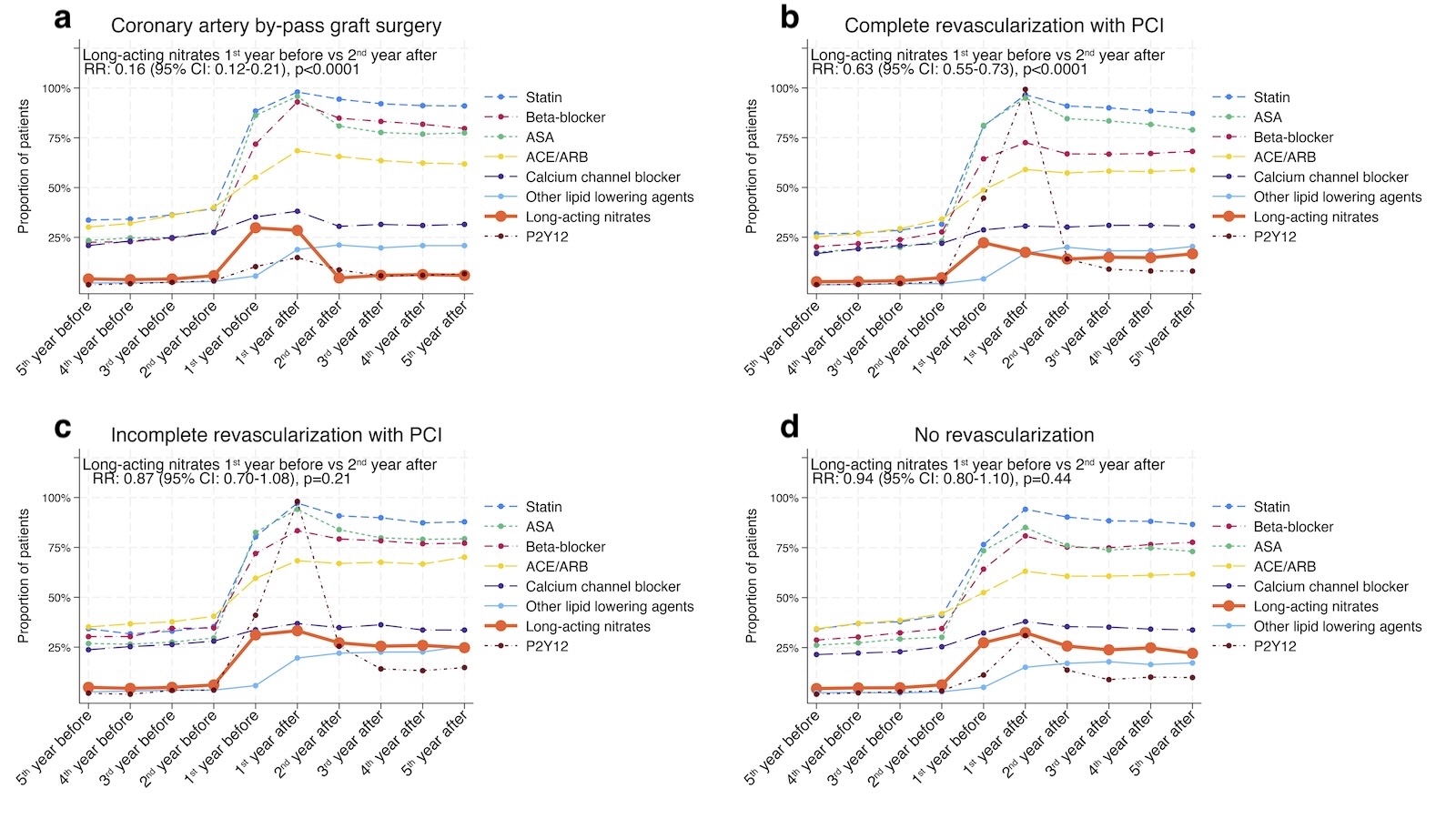
**

**Supplementary Figure 7. Sensitivity analysis including only patients admitted to university hospitals,** this analysis including only patients admitted to university hospitals (n=7). A total of 5183 patients were eligible for this analysis. Use of long-acting nitrates was compared between the periods 1 year to angiography and 1-2 year after angiography and was assessed using univariable conditional Poisson regression. Results are presented as risk-ratios (RR) along with 95% confidence interval (CI) together with p-value.

ACE = angiotensin converting enzyme inhibitor; ARB = angiotensin receptor blocker; ASA = acetylsalicylic acid; CI = confidence interval; PCI = percutaneous coronary intervention; RR = risk-ratio.
